# Aseptic and Physical Means of Treatment for War Wounds in Various Stages

**Published:** 1917-12

**Authors:** 


### TREATMENT

***Aseptic and Physical Means of Treatment for War Wounds in Various Stages.*** By R. LERICHE, Médecin-Major. Trans. and abs. from the *Lyon Chirurgical*, January-February, 1916.

L. undertakes to establish :

1. That immediate mechanical cleansing of war wounds suffices, *without the use of antiseptics*, to insure the arrest of serious infections, and normal evolution towards healing; this is accomplished without any suppuration if the operation is very early, and with only slight suppuration if the operation is performed reasonably soon.

2. That the opening of infected wounds by means of large excisions, and with immediate removal of all foreign bodies remaining in place, and of *organic gangrenous matter*, is sufficient, *without antiseptics* to arrest, the infectious processes, and permits of healing as early as is usual in civil cases.

3. That disinfection and the repair and healing of infected wounds are accelerated by the early and methodical use of the physical agents of sun, hot air, etc., *without any recourse to chemical preparations.*

4. That in chronic infections of long standing, such as fistulae or other results of infection, and in delayed healing of the soft parts against which antiseptics are of no avail, active surgical treatment permits of rapid healing by reason of the removal of foreign bodies, of sequestra, and of bony cavities, as well as by skin grafts and the use of large autoplastic subcutaneous detachments.

In short, the author declares : " *Opening to the air in the fashion of Poncet, by means of a generous and purely aseptic operation, aided from the start by physical agents, is the best manner of treatment for war wounds in all stages of their development : these wounds are not, at any time, amenable to chemico-therapy; they arise in mechanical disturbances and their treatment should be physiotherapy.* "

At the start the operator should regard every wound as suspicious if not actually infected, and, in every case, enlarge it, cleanse it mechanically, and expose it to the open air, according to former theories of primary cleansing, such as would control treatment in civil cases. In civil practice all the surgical principles necessary in the treatment of war wounds have long since been known and put into practice. L. believes that if war surgeons like civil surgeons will open deep infected pockets, remove all centers of infection, and provide for adequate drainage, there will be no larger proportion of deaths from infections in war than in times of peace.

Such treatment, the author believes, is especially effective in dealing with *gaseous gangrene*. The three wound conditions most to be feared in this type of infection are : *destruction of muscle, the presence of foreign bodies, and hidden pockets.* Since gas gangrene is exclusively a muscle infection, and since it arises not from the mere presence of the organism, but from certain conditions in the wound, especially necrosis, which favor the fatal activity of the bacteria, the only sure way of dealing with this infection is complete excision of the affected muscle. It suffices, however, to remove only the toxic and gangrenous tissue with the whole zone of the infection. Generally the infectious process is confined to a single muscle, although the gas may have extended far beyond.

In case of injury to the blood vessels, L. urges removal of all hematoma, and the application of ligatures to the vessels so as to leave no media for infection in the wound.

With regard to the bones, L. insists upon the removal " not only of free bone fragments in the wound, and of bone splinters on which bits of garments are sometimes found, but also of adherent splinters with a vital periosteum, the health of which might be endangered by infection, and which might hinder the ventilation of the fracture. "

The author reviews with considerable detail his own extended experience in dealing with war wounds. Out of about 2,000 limb wounds treated according to these principles practically no cases of serious infection occurred, except when the operation was incomplete, or certain fragments of foreign matter were overlooked. He believes that the surgeon " ought not to proceed according to immediate clinical indications of infection, but with a view to the possibilities of its development. "

By way of post-operative treatment he urges recourse first of all to heliotherapy, and testifies to the remarkable and immediate results of this method of dealing with infected surfaces which are properly exposed. If sun treatment is impossible, hot air and artificial light methods, although not so effective, are sure to give good results.

